# Supplementary material for: Role of immunosuppression in an antibiotic stewardship intervention and its association with clinical outcomes and antibiotic use: protocol for an observational study (RISC-sepsis)
Source: BMJ Open. 2022 Dec 9;12(12):e068321. doi: 10.1136/bmjopen-2022-068321 (PMC9743405; doi:10.1136/bmjopen-2022-068321)
Supplement: Author's manuscript [file bmjopen-2022-068321.draft_revisions.pdf]

BMJ Open is committed to open peer review. As part of this commitment we make the peer review history of every article we publish publicly available.

When an article is published we post the peer reviewers' comments and the authors' responses online. We also post the versions of the paper that were used during peer review. These are the versions that the peer review comments apply to.

The versions of the paper that follow are the versions that were submitted during the peer review process. They are not the versions of record or the final published versions. They should not be cited or distributed as the published version of this manuscript.

BMJ Open is an open access journal and the full, final, typeset and author-corrected version of record of the manuscript is available on our site with no access controls, subscription charges or pay-per-view fees (http://bmjopen.bmj.com).

If you have any questions on BMJ Open's open peer review process please email info.bmjopen@bmj.com

BMJ Open

The Role of Immunosuppression in an antibiotic Stewardship intervention and its association with Clinical outcomes and antibiotic use: Protocol for an observational study (RISC-sepsis)

Journal:	*BMJ Open*
Manuscript ID	bmjopen-2022-068321
Article Type:	Protocol
Date Submitted by the Author:	14-Sep-2022
Complete List of Authors:	Scott, Jonathan; Newcastle University, Translational and Clinical Research Institute<br>Trevi, Loredana; Newcastle University, Translational and Clinical Research Institute<br>McNeil, Hannah; University of Warwick, Clinical Trials Unit<br>Ewen, Tom; Newcastle University, Translational and Clinical Research Institute<br>Mawson, Phil; Newcastle University, Translational and Clinical Research Institute<br>McDonald, David; Newcastle University, Flow Cytometry Core Facility<br>Filby, Andrew; Newcastle University, Flow Cytometry Core Facility<br>Lall, Ranjit; University of Warwick, Warwick Clinical Trials Unit<br>Booth, Katie; University of Warwick, Warwick Clinical Trials Unit<br>Boschman, Gert; Becton Dickinson<br>Melkebeek, Vesna; Becton Dickinson<br>Perkins, Gavin; University of Warwick, Clinical Trials Unit<br>McMullan, Ronan; Royal Victoria Hospital, Department of Medical Microbiology; Queen's University Belfast, Wellcome Wolfson Institute for Experimental Medicine<br>McAuley, Daniel; Queen's University Belfast, Centre for Experimental Medicine; Belfast Health and Social Care Trust, Regional Intensive Care Unit<br>McCullagh, Iain J; Newcastle Upon Tyne Hospitals NHS Foundation Trust, Department of Perioperative medicine; Freeman Hospital, Department of Perioperative medicine<br>Walsh, Timothy; Edinburgh Royal Infirmary, Intensive Care Unit; The University of Edinburgh, The Queen's Medical Research Institute<br>Rostron, Anthony; South Tyneside and Sunderland NHS Foundation Trust, Integrated Critical Care Unit; Newcastle University, Translational and Clinical Research Institute<br>Shankar-Hari, Manu; University of Edinburgh Division of Clinical and Surgical Sciences, Centre for Inflammation Research; Edinburgh Royal Infirmary, Intensive Care Unit<br>Dark, Paul; University of Manchester, Division of Immunology; Salford Care Organisation, Critical Care Department<br>Simpson, A John; Newcastle University Institute of Cellular Medicine, Institute of Cellular Medicine, Medical School; Royal Victoria Infirmary, Department of Respiratory Medicine

Conway-Morris, Andrew; University of Cambridge, Department of Medicine; Addenbrooke's Hospital, JVF Intensive Care Unit
Hellyer, Thomas P; Newcastle University, Translational and Clinical Research Institute; Royal Victoria Infirmary, Department of Critical Care Medicine

Keywords: Adult intensive & critical care < ANAESTHETICS, Immunology < NATURAL SCIENCE DISCIPLINES, MICROBIOLOGY

SCHOLARONE™ Manuscripts

The Role of Immunosuppression in an antibiotic Stewardship intervention and its association with Clinical outcomes and antibiotic use: Protocol for an observational study (RISC-sepsis)

Jonathan Scott[1†], Loredana Trevi[1†], Hannah McNeil[2], Tom Ewen[1], Phil Mawson[1], David McDonald[3], Andrew Filby[3], Ranjit Lall[2], Katie Booth[2], Gert Boschman[4], Vesna Melkebeek[4], Gavin D Perkins[2,5], Ronan McMullan[6, 7], Danny McAuley[6, 8], Iain J McCullagh [1, 9], Timothy S Walsh[10, 11], Anthony Rostron[1, 12], Manu Shankar-Hari[10, 11], Paul Dark[13, 14], A John Simpson[1, 15], Andrew Conway Morris[16, 17,18], Thomas P Hellyer[1, 19*]

[†]These authors contributed equally

*Corresponding author

1. Translational and Clinical Research Institute, Faculty of Medical Sciences, Newcastle University, Newcastle-upon-Tyne, NE2 4HH
2. Warwick Clinical Trials Unit, Warwick Medical School, The University of Warwick, Coventry, CV4 7AL
3. Flow Cytometry Core Facility, Faculty of Medical Sciences, Newcastle University, Newcastle-upon-Tyne, NE2 4HH
4. Becton Dickinson, Erembodegem, Belgium
5. Critical Care Department, Birmingham Heartlands Hospital, Birmingham, B9 5SS
6. Wellcome Wolfson Institute for Experimental Medicine, Queens University Belfast, University Road, Belfast, BT7 1NN
7. Microbiology Department, Royal Victoria Hospital, Belfast, BT12 6BA
8. Regional Intensive Care Unit, Royal Victoria Hospital, Belfast Health and Social Care, Belfast, BT12 6BA
9. Department of Perioperative medicine, Critical care and Pain, Freeman Hospital, Newcastle-upon-Tyne, NE7 7DN
10. The Queen's Medical Research Institute, Edinburgh BioQuarter, Centre for Inflammation Research, Edinburgh, EH16 4TJ
11. Intensive Care Unit, Edinburgh Royal Infirmary, Edinburgh, EH16 4SA
12. Integrated Critical Care Unit, Sunderland Royal Hospital, South Tyneside and Sunderland NHS Foundation Trust, Sunderland, SR4 7TP
13. Division of Immunology, Immunity to Infection and Respiratory Medicine, Faculty of Biology Medicine and Health, The University of Manchester, Manchester, M13 9PL
14. Critical Care Department, Northern Care Alliance NHS Foundation Trust, Salford Care Organisation, Greater Manchester, M6 8HD
15. Department of Respiratory Medicine, Royal Victoria Infirmary, Newcastle, NE1 4LP
16. Division of Anaesthesia, Department of Medicine, University of Cambridge, Cambridge, CB2 0QQ
17. Division of Immunology, Department of Pathology, University of Cambridge, Cambridge, CB2 1QP
18. JVF Intensive Care Unit, Addenbrooke's Hospital, Cambridge, CB2 0QQ
19. Department of Critical Care Medicine, Royal Victoria Infirmary, Newcastle, NE1 4LP

For peer review only

Abstract

Introduction

Sepsis is characterised by a dysregulated immune response to infection, with exaggerated pro-inflammatory and anti-inflammatory responses. A predominant immunosuppressive profile affecting both innate and adaptive immune responses is associated with increased hospital-acquired infection and reduced infection-free survival. While hospital-acquired infection leads to additional antibiotic use, the role of the immunosuppressive phenotype in guiding complex decisions, such as those affecting antibiotic stewardship, is uncertain. This study is a mechanistic sub-study embedded within a multi-centre clinical and cost-effectiveness trial of biomarker-guided antibiotic stewardship, which aims to determine the effect of sepsis-associated immunosuppression on antibiotic use.

Methods and analysis

RISC-sepsis is a prospective, multi-centre, exploratory, observational study embedded within the ADAPT-Sepsis trial. A sub-group of 180 participants with antibiotics commenced for suspected sepsis, enrolled in the ADAPT-sepsis trial, will be recruited. Blood samples will be collected on alternate days until day 7. At each time point, blood will be collected for flow cytometric analysis into cell preservation tubes. Immunophenotyping will be performed at a central testing hub by flow cytometry. The primary outcome measures are monocyte HLA-DR; neutrophil CD88; programmed cell death-1 (PD-1) on monocytes, neutrophils and T lymphocytes; and the percentage of regulatory T cells. Secondary outcome measures will link to trial outcomes from the ADAPT-Sepsis trial including antibiotic days; occurrence of hospital-acquired infection; and length of ICU- and hospital-stay.

Ethics and dissemination

Ethical approval has been granted (IRAS 209815) and the study registered with the ISRCTN (86837685). Study results will be disseminated by peer-reviewed publications, presentations at scientific meetings, and via patient and public participation groups and social media.

Strengths and weaknesses

- Multi-centre study recruiting a broad cohort of patients representative of critically ill patients with sepsis in the UK.
- Centralised flow cytometry immunophenotyping using preservation methods allowing standardisation in the context of multi-centre recruitment.
- Embedded within a clinical trial, immunophenotypes will be linked to robust clinical outcomes.
- As an observational study RISC-sepsis will provide insights into the impact of sepsis-associated immunosuppression on a biomarker-guided antibiotic duration intervention but since it is not interventional, it will not offer a robust precision-medicine approach to antibiotic stewardship.

Introduction

Sepsis is a syndrome of life-threatening organ dysfunction secondary to a dysregulated immune response to an infection (1). Mortality from sepsis is high, with 31% of patients dying in hospital and a further 15% of survivors dying in the subsequent year (2,3). As a leading cause of death, there are

For peer review only

an estimated 31.5 million cases of sepsis worldwide each year (4). Organ support in a critical care setting is often required and patients with sepsis account for a third of general adult critical care admissions in the UK (2).

The dysregulated immune response that characterises sepsis is a paradigm of both a pro-inflammatory and an anti-inflammatory response (5,6). While these immune states can occur simultaneously, a predominant immunosuppressive profile has been associated with hospital-acquired infections and mortality (7,8). This sepsis-associated immunosuppression is demonstrated across both the innate and adaptive immune responses with defects in monocyte, neutrophil and T cell function (9–12). The persistence of these cellular abnormalities and the accumulation of dysfunctions increase the risk of adverse clinical outcomes (10,13–15).

While an immunosuppressive profile been linked to poor clinical outcomes, the influence that it has in guiding complex clinical decision-making processes, such as those involved in antibiotic stewardship remains uncertain. Antibiotic treatment in sepsis may be life-saving but the overuse of antibiotics leads to the emergence of antimicrobial resistance (AMR) and adverse clinical outcomes (16–19). The optimal duration of antibiotic treatment in sepsis is unknown (20). Biomarker-guided antibiotic durations are not recommended for routine use in the UK (21) and the current evidence for effectiveness is regarded as low quality internationally (22). The ADAPT-Sepsis trial (ISRCTN47473244) is a UK National Institute for Health and Care Research (NIHR) Health Technology Assessment-commissioned clinical trial that will evaluate the clinical and cost effectiveness of procalcitonin (PCT) and C-reactive protein (CRP) protocols for biomarker-guided antibiotic duration decisions in a National Health Service (NHS) setting.

This protocol outlines a mechanistic study embedded within the ADAPT-Sepsis trial. RISC-sepsis will immune phenotype patients using leukocyte surface biomarkers and dichotomise patients into two groups, 'sepsis-associated immunosuppression' and 'non-immunosuppressed' (definition below). We will then explore the effectiveness of the biomarker-guided antibiotic duration intervention and the impact on trial outcomes for patients with sepsis-associated immunosuppression.

Rationale

The ADAPT-Sepsis trial will include a heterogeneous population of adult patients with sepsis, including those with sepsis-induced immunosuppression. As a phenotype associated with hospital-acquired infections and reduced infection-free survival, there may be heterogeneity in the effect of the biomarker-guided antibiotic duration intervention for patients with this phenotype. If biomarker-guided antibiotics are ineffective in patients with sepsis-associated immunosuppressed, then a greater degree of patient stratification may be required to ensure optimal antibiotic duration. Furthermore, if CRP and PCT levels fall over the same period that cellular defects resolve, this would give clinicians greater confidence in the biomarker-guided antibiotic durations.

Research question

Does sepsis-associated immunosuppression render a PCT/CRP biomarker-guided antibiotic duration intervention ineffective?

Hypothesis

For peer review only

Sepsis-associated immunosuppression will be associated with: longer duration of antibiotics, due to persistently raised PCT and CRP; more hospital-acquired infection; and more antibiotic treatment days.

Primary objective

To determine differences in ADAPT-sepsis trial outcomes between patients defined as 'sepsis-associated immunosuppressed' versus 'non-immunosuppressed'.

Secondary objective

To monitor changes in expression of leukocyte surface markers (primary outcome measures) over the time course of the ADAPT-Sepsis biomarker-guided antibiotic duration intervention period.

Methods and analysis

RISC-sepsis is a prospective, multi-centre, exploratory, observational study embedded within the ADAPT-Sepsis trial.

Population

We will include sequential patients who are enrolled into the ADAPT-sepsis trial within sites in the RISC-sepsis sub-study. Patients are included in the ADAPT-sepsis trial based on the following inclusion criteria:

1. Hospitalised adult patients at least 18 years of age
2. Up to 24 hours since initiation of antibiotics for suspected sepsis
3. Likely to remain hospitalised and requiring intravenous antibiotics for the next 72 hours
4. Requirement for critical care

Patients are excluded based on the following criteria:

1. More than 24 hours since receiving first empiric intravenous antibiotic treatments for suspicion of sepsis
2. Prolonged (greater than 21 days) antimicrobial therapy mandated (e.g. for endocarditis, cerebral/hepatic abscess, tuberculosis, osteomyelitis)
3. Severely immunocompromised (e.g. blood neutrophil count less than 0.5 x 10^9/L) not caused by sepsis
4. Any patient given, or anticipated to receive an IL-6 receptor inhibitor drug (e.g. tocilizumab or sarilumab) during their acute hospital admission
5. All treatment for suspected sepsis likely to be stopped within 24 hours of its initiation because of futility
6. Patient participation declined
7. Previously enrolled in ADAPT-Sepsis

Outcomes

For peer review only

The primary outcomes are markers of blood leukocyte function measured by flow cytometry. These primary markers are:

- Monocyte HLA-DR
- Neutrophil CD88
- Programmed cell death-1 on T lymphocytes (CD4-positive and CD8-positive), monocytes and neutrophils
- Percentage of regulatory T cells

Secondary outcome measures include outcome measures derived from the ADAPT-Sepsis trial including duration of antibiotics during the ADAPT-Sepsis intervention phase; total antibiotic days measured at 28 days; occurrence of hospital-acquired infection; and length of ICU- and hospital-stay. Secondary measures will also include PCT and CRP concentrations in serum.

Sampling and data collection

As a sub-study within the ADAPT-Sepsis trial, all RISC-sepsis procedures and sampling will occur within the trial. Sampling will occur during the ADAPT-Sepsis intervention period when blood is sampled daily. While additional blood samples are taken from patients for RISC-sepsis, there is no increase in number of sampling episodes. Patients will have blood sampled on alternate days up to 4 sampling points (days 1, 3, 5 and 7). Blood for flow cytometric analysis will be collected in Cyto-Chex (Streck, La Vista, USA) blood preservation tubes before shipping to a central testing hub (Newcastle University). Serum will be collected, frozen and stored at site until the end of the recruitment period.

Baseline clinical data and outcome data will be obtained from the ADAPT-sepsis data collection. Baseline data will include admission diagnosis, site of infection, acute physiology and chronic health evaluation (APACHE) II and sequential organ failure assessment (SOFA) scores, and sepsis bundle care elements in the first 24 hours. In addition, at each RISC-sepsis sampling point, the circulating white blood cell count and differential count will be recorded.

Blood sample analysis

Flow cytometric analysis will be performed on a single centralised (Newcastle University) BD FACSymphony A5 flow cytometer (Becton Dickinson Biosciences, San Jose, California, USA, from here BDB). Daily internal quality control will be performed using Cytometry Setup and Tracking beads (BDB).

Leukocyte staining will be performed using antibody:fluorophore conjugates supplied by BDB. Samples will be processed according to a standard operating procedure.

At each time point, serum will be collected for measurement of PCT and CRP. These will be batched and processed by enzyme-linked immunosorbent assay (ELISA) at the end of the recruitment period.

Immune phenotyping

Cells will be identified based on light scatter properties and cellular markers: CD15++ neutrophils; CD14+ monocytes; CD3+ CD4+ T cells; CD3+ CD8+ T cells; and CD3+ CD4+ CD25+ CD127lo

For peer review only

regulatory T cells. Expression of monocyte HLA-DR, neutrophil CD88 and PD-1 (CD279) on monocytes, neutrophils and T lymphocytes will be measured.

In addition, exploratory analyses will include: CD14+ CD16- classical monocytes; CD14+ CD16+ intermediate monocytes; CD14- CD16+ non-classical monocytes; CD3- CD19+ B cells; CD3- CD56+ NK cells; CD3- CD19- CD14- CD16- CD56- HLA-DR+ dendritic cells. In addition to expression of HLA-DR and CD279 in cell populations, CD274 (PD-L1) will also be measured.

Data will be assessed for evidence of batch effects and normalisation approaches will be used. Expert manual gating will be performed using a pre-defined gating standard operating procedure. We will report number and percentage of cell populations in blood and median fluorescent intensity of key markers.

Definition of sepsis-associated immunosuppression

We will define 'sepsis-associated immunosuppression' as two or more abnormalities (from low HLA-DR, low CD88, raised PD-1 or raised percentage regulatory T cells) that persist for 3 or more days (14,23). Previous reports have shown that the persistence of immune dysfunctions is associated with worse clinical outcome including increased hospital-acquired infections and death (10,13,15). The 'non-immunosuppressed' group will be patients that do not meet this criteria.

Sample size

The sample size is based on detection of a difference in antibiotic durations between the 'sepsis-associated immunosuppression' and 'non-immunosuppressed' groups. The 2016 Surviving Sepsis Campaign guidelines recommended 7-10 days of antibiotics for patients with sepsis (24). We have assumed that patients with sepsis-associated immunosuppression will have longer durations (10 days) and so with a standard deviation of 6 days (derived from the ADAPT-sepsis trial), then a sample of 180 patients is required to detect a difference of 3 days in antibiotic treatment between the 'sepsis-associated immunosuppressed' and 'non-immunosuppressed' patients with 90% power, a 5% alpha and a 5% withdrawal rate.

Data analysis plan

A formal analysis plan will be finalised and deposited on an open access platform before the database is locked.

For the primary objective, the time it takes for antibiotics to be stopped in patients during the ADAPT-sepsis biomarker-guided antibiotic phase will be summarised and the distribution of the outcome described for the 'sepsis-associated immunosuppression' and the 'non-immunosuppressed' groups. The mean number of antibiotic days will be compared between groups obtained from linear regression models and adjusted for confounding variables. In addition, each of the cellular markers will be assessed as a continuous outcome. The change in cellular markers between each time point will be correlated with the number of days on antibiotics using Spearman's correlation coefficient. One sample t-tests will be used to assess the changes between time points.

The trial outcomes of 28-day antibiotics, hospital-acquired infections and length of ICU- and hospital-stay will be summarised for the 'sepsis-associated immunosuppressed' and 'non-immunosuppressed'

For peer review only

groups. Differences between groups will be assessed using linear regression for continuous variables and logistic regression for categorical variables. We will describe the difference between the groups using p-values, point estimates and 95% confidence intervals.

For the secondary objective, differences in PCT and CRP levels (point estimate and 95% confidence interval) between periods when patients met criteria for 'sepsis-associated immunosuppressed' and when immune profile recovered (i.e. became 'non-immunosuppressed'). This will be performed by identifying the time interval during which the patients were immunosuppressed and, within this time interval, we shall assess the PCT/CRP levels (averaged over time for each patient). A comparison will be made with the time periods when the same patient was not immunosuppressed. Linear regression models will be used to make a comparison of the two states within patients, with the patient as a random effect (to account for within-patient level). Percentage change in cellular markers over time will be compared to change in CRP and PCT over time.

Sub-group analyses:

Exploratory analysis will be carried out for the following sub-groups:

1. Patients receiving critical care for a medical *versus* a surgical cause of sepsis.
2. Patients receiving critical care because of trauma *versus* non-trauma.
3. Patients receiving critical care because of a community-acquired infection *versus* a hospital-acquired infection
4. Infection site: community-acquired pneumonia; hospital-acquired pneumonia; urinary tract infection; intra-abdominal infection; or positive blood culture.
5. Patients with septic shock.

Regression models will be used with 'sepsis-associated immunosuppressed' as an interaction term by sub-group for number of days on antibiotics.

Public and Patient Involvement

Through Pathfinder, a patient representatives and critical care survivors group in the North East of England, public and patients were involved in the study design. Ongoing public and patient involvement is through the ADAPT-Sepsis Trial Management and Trial Steering Groups, which incorporate RISC-sepsis oversight, and have members of the Intensive Care Society's Patient and Relatives group.

Ethics and dissemination

As a sub-study, the protocol has been incorporated into the ADAPT-sepsis protocol and ethical approval has been granted by the South Central – Oxford C Research Ethics Committee (IRAS 209815). The study has been registered with the ISRCTN registry (86837685) and adopted by the UK National Institute for Health and Care Research (NIHR) Clinical Research Portfolio. The study is managed by the Warwick Clinical Trials Unit (WCTU) and Sponsored by The University of Manchester. Independent study oversight is provided by the ADAPT-Sepsis trial steering committee.

The study will be reported in line with the STROBE guidelines for observational studies (25). Findings will be disseminated by peer-reviewed publications and at national and international

For peer review only

meetings. In addition, findings will be disseminated via public and patient groups and through social media.

References

1. Singer M, Deutschman CS, Seymour C, Shankar-Hari M, Annane D, Bauer M, et al. The third international consensus definitions for sepsis and septic shock (sepsis-3). JAMA. 2016;315(8):801–10.
2. Shankar-Hari M, Harrison DA, Rubenfeld GD, Rowan K. Epidemiology of sepsis and septic shock in critical care units: comparison between sepsis-2 and sepsis-3 populations using a national critical care database. Br J Anaesth. 2017 Oct 1;119(4):626–36.
3. Shankar-Hari M, Harrison DA, Ferrando-Vivas P, Rubenfeld GD, Rowan K. Risk Factors at Index Hospitalization Associated With Longer-term Mortality in Adult Sepsis Survivors. JAMA Netw Open. 2019;2(5):e194900.
4. Fleischmann C, Scherag A, Adhikari NKJ, Hartog CS, Tsaganos T, Schlattmann P, et al. Assessment of Global Incidence and Mortality of Hospital-treated Sepsis. Current Estimates and Limitations. Am J Respir Crit Care Med. 2016;193(3):259–72.
5. van der Poll T, van de Veerdonk FL, Scicluna BP, Netea MG. The immunopathology of sepsis and potential therapeutic targets. Nat Rev Immunol. 2017;17(7):407–20.
6. Hotchkiss RS, Monneret G, Payen D. Immunosuppression in sepsis: a novel understanding of the disorder and a new therapeutic approach. Lancet Infect Dis. 2013;13(3):260–8.
7. Boomer JS, To K, Chang KC, Takasu O, Osborne DF, Walton AH, et al. Immunosuppression in patients who die of sepsis and multiple organ failure. JAMA. 2011;306(23):2594–605.
8. Pfortmueller CA, Meisel C, Fux M, Schefold JC. Assessment of immune organ dysfunction in critical illness: utility of innate immune response markers. Intensive Care Med Exp. 2017;5(1):49.
9. Guignant C, Lepape A, Huang X, Kherouf H, Denis L, Poitevin F, et al. Programmed death-1 levels correlate with increased mortality, nosocomial infection and immune dysfunctions in septic shock patients. Crit Care. 2011;15(2):R99.
10. Cheron A, Floccard B, Allaouchiche B, Guignant C, Poitevin F, Malcus C, et al. Lack of recovery in monocyte human leukocyte antigen-DR expression is independently associated with the development of sepsis after major trauma. Crit Care. 2010;14(6):R208.
11. Conway Morris A, Kefala K, Wilkinson TS, Dhaliwal K, Farrell L, Walsh T, et al. C5a mediates peripheral blood neutrophil dysfunction in critically ill patients. Am J Respir Crit Care Med. 2009;180(1):19–28.
12. Zhao Y, Jia Y, Li C, Fang Y, Shao R. The risk stratification and prognostic evaluation of soluble programmed death-1 on patients with sepsis in emergency department. Am J Emerg Med. 2018;36(1):43–8.
13. Bodinier M, Peronnet E, Brengel-Pesce K, Conti F, Rimmelé T, Textoris J, et al. Monocyte Trajectories Endotypes Are Associated With Worsening in Septic Patients. Front Immunol. 2021;12:5096.
14. Conway Morris A, Datta D, Shankar-Hari M, Stephen J, Weir CJ, Rennie J, et al. Cell-surface signatures of immune dysfunction risk-stratify critically ill patients: INFECT study. Intensive Care Med. 2018;44(5):627–35.
15. Drewry AM, Samra N, Skrupky LP, Fuller BM, Compton SM, Hotchkiss RS. Persistent lymphopenia after diagnosis of sepsis predicts mortality. Shock. 2014;42(5):383–91.
16. Chatterjee A, Modarai M, Naylor NR, Boyd SE, Atun R, Barlow J, et al. Quantifying drivers of antibiotic resistance in humans: a systematic review. Lancet Infect Dis. 2018;18(12):e368–

For peer review only

78.
17. Bonnet V, Dupont H, Glorion S, Aupée M, Kipnis E, Gérard JL, et al. Influence of bacterial resistance on mortality in intensive care units: a registry study from 2000 to 2013 (IICU Study). J Hosp Infect. 2019;102(3):317–24.
18. Kett DH, Cano E, Quartin AA, Mangino JE, Zervos MJ, Peyrani P, et al. Implementation of guidelines for management of possible multidrug-resistant pneumonia in intensive care: an observational, multicentre cohort study. Lancet Infect Dis. 2011;11(3):181–9.
19. Tamma PD, Avdic E, Li DX, Dzintars K, Cosgrove SE. Association of Adverse Events With Antibiotic Use in Hospitalized Patients. JAMA Intern Med. 2017;177(9):1308.
20. Hellyer TP, Mantle T, McMullan R, Dark P. How to optimise duration of antibiotic treatment in patients with sepsis? BMJ. 2020;371:11–3.
21. NICE. Procalcitonin testing for diagnosing and monitoring sepsis (ADVIA Centaur BRAHMS PCT assay, BRAHMS PCT Sensitive Kryptor assay, Elecsys BRAHMS PCT assay, LIAISON BRAHMS PCT assay and VIDAS BRAHMS PCT assay) DG18. 2015.
22. Evans L, Rhodes A, Alhazzani W, Antonelli M, Coopersmith CM, French C, et al. Surviving Sepsis Campaign: International Guidelines for Management of Sepsis and Septic Shock 2021. Crit Care Med. 2021;49(11):e1063–143.
23. Shankar-Hari M, Datta D, Wilson J, Assi V, Stephen J, Weir CJ, et al. Early PREdiction of sepsis using leukocyte surface biomarkers: the ExPRES-sepsis cohort study. Intensive Care Med. 2018;44(11):1836–1848.
24. Rhodes A, Evans LE, Alhazzani W, Levy MM, Antonelli M, Ferrer R, et al. Surviving Sepsis Campaign: International Guidelines for Management of Sepsis and Septic Shock: 2016. Crit Care Med. 2017;45(3):486–552.
25. Vandenbroucke JP, Von Elm E, Altman DG, Gøtzsche PC, Mulrow CD, Pocock SJ, et al. Strengthening the Reporting of Observational Studies in Epidemiology (STROBE): Explanation and Elaboration. PLOS Med. 2007;4(10):e297.

For peer review only

Authors' contribution

Study conception: TPH, ACM, MSH, PD, TW, GB
Acquisition of funding: TPH, ACM, MSH, PD, RMcM, DFMcA, TW, AJS, TR, GP
Development of flow cytometry methods: JS, LT, DFMcA, AF, TR, ACM, MSH, GB, VM
Project management: HMcN, TE, PM, TPH
Statistical analysis: RL, TPH
Writing - draft manuscript: TPH, ACM, AJS, AF
Writing – reviewing and editing: All authors contributed

Funding statement

This study/project is funded by the NIHR Efficacy and Mechanism Evaluation (EME) (128374). The views expressed are those of the author(s) and not necessarily those of the NIHR or the Department of Health and Social Care. Regents for flow cytometric analysis are provided by Becton Dickinson Biosciences. The study is also supported by the MRC SHIELD consortium (MRNO2995X/1).

Competing interests statement

DFMcA is NIHR/MRC EME programme director and has previously sat on NIHR HTA funding committees.

All other authors report no competing interests in relation to this work.

For peer review only

BMJ Open

The Role of Immunosuppression in an antibiotic Stewardship intervention and its association with Clinical outcomes and antibiotic use: Protocol for an observational study (RISC-sepsis)

Journal:	*BMJ Open*
Manuscript ID	bmjopen-2022-068321.R1
Article Type:	Protocol
Date Submitted by the Author:	14-Nov-2022

Complete List of Authors:	Scott, Jonathan; Newcastle University, Translational and Clinical Research Institute<br>Trevi, Loredana; Newcastle University, Translational and Clinical Research Institute<br>McNeil, Hannah; University of Warwick, Clinical Trials Unit<br>Ewen, Tom; Newcastle University, Translational and Clinical Research Institute<br>Mawson, Phil; Newcastle University, Translational and Clinical Research Institute<br>McDonald, David; Newcastle University, Flow Cytometry Core Facility<br>Filby, Andrew; Newcastle University, Flow Cytometry Core Facility<br>Lall, Ranjit; University of Warwick, Warwick Clinical Trials Unit<br>Booth, Katie; University of Warwick, Warwick Clinical Trials Unit<br>Boschman, Gert; Becton Dickinson<br>Melkebeek, Vesna; Becton Dickinson<br>Perkins, Gavin; University of Warwick, Clinical Trials Unit<br>McMullan, Ronan; Royal Victoria Hospital, Department of Medical Microbiology; Queen's University Belfast, Wellcome Wolfson Institute for Experimental Medicine<br>McAuley, Daniel; Queen's University Belfast, Centre for Experimental Medicine; Belfast Health and Social Care Trust, Regional Intensive Care Unit<br>McCullagh, Iain J; Newcastle Upon Tyne Hospitals NHS Foundation Trust, Department of Perioperative medicine; Freeman Hospital, Department of Perioperative medicine<br>Walsh, Timothy; Edinburgh Royal Infirmary, Intensive Care Unit; The University of Edinburgh, The Queen's Medical Research Institute<br>Rostron, Anthony; South Tyneside and Sunderland NHS Foundation Trust, Integrated Critical Care Unit; Newcastle University, Translational and Clinical Research Institute<br>Shankar-Hari, Manu; University of Edinburgh Division of Clinical and Surgical Sciences, Centre for Inflammation Research; Edinburgh Royal Infirmary, Intensive Care Unit<br>Dark, Paul; University of Manchester, Division of Immunology; Salford Care Organisation, Critical Care Department<br>Simpson, A John; Newcastle University Institute of Cellular Medicine, Institute of Cellular Medicine, Medical School; Royal Victoria Infirmary, Department of Respiratory Medicine

	Conway-Morris, Andrew; University of Cambridge, Department of Medicine; Addenbrooke's Hospital, JVF Intensive Care Unit<br>Hellyer, Thomas P; Newcastle University, Translational and Clinical Research Institute; Royal Victoria Infirmary, Department of Critical Care Medicine

**Primary Subject Heading**:	Intensive care
Secondary Subject Heading:	Infectious diseases, Immunology (including allergy), Diagnostics
Keywords:	MICROBIOLOGY, INTENSIVE & CRITICAL CARE, INFECTIOUS DISEASES

SCHOLARONE™ Manuscripts

The Role of Immunosuppression in an antibiotic Stewardship intervention and its association with Clinical outcomes and antibiotic use: Protocol for an observational study (RISC-sepsis)

Jonathan Scott[1†], Loredana Trevi[1†], Hannah McNeil[2], Tom Ewen[1], Phil Mawson[1], David McDonald[3], Andrew Filby[3], Ranjit Lall[2], Katie Booth[2], Gert Boschman[4], Vesna Melkebeek[4], Gavin D Perkins[2,5], Ronan McMullan[6, 7], Danny McAuley[6, 8], Iain J McCullagh [1, 9], Timothy S Walsh[10, 11], Anthony Rostron[1, 12], Manu Shankar-Hari[10, 11], Paul Dark[13, 14], A John Simpson[1, 15], Andrew Conway Morris[16, 17,18], Thomas P Hellyer[1, 19*]

[†]These authors contributed equally

*Corresponding author, thomas.hellyer@newcastle.ac.uk

1. Translational and Clinical Research Institute, Faculty of Medical Sciences, Newcastle University, Newcastle-upon-Tyne, NE2 4HH
2. Warwick Clinical Trials Unit, Warwick Medical School, The University of Warwick, Coventry, CV4 7AL
3. Flow Cytometry Core Facility, Faculty of Medical Sciences, Newcastle University, Newcastle-upon-Tyne, NE2 4HH
4. Becton Dickinson, Erembodegem, Belgium
5. Critical Care Department, Birmingham Heartlands Hospital, Birmingham, B9 5SS
6. Wellcome Wolfson Institute for Experimental Medicine, Queens University Belfast, University Road, Belfast, BT7 1NN
7. Microbiology Department, Royal Victoria Hospital, Belfast, BT12 6BA
8. Regional Intensive Care Unit, Royal Victoria Hospital, Belfast Health and Social Care, Belfast, BT12 6BA
9. Department of Perioperative medicine, Critical care and Pain, Freeman Hospital, Newcastle-upon-Tyne, NE7 7DN
10. The Queen's Medical Research Institute, Edinburgh BioQuarter, Centre for Inflammation Research, Edinburgh, EH16 4TJ
11. Intensive Care Unit, Edinburgh Royal Infirmary, Edinburgh, EH16 4SA
12. Integrated Critical Care Unit, Sunderland Royal Hospital, South Tyneside and Sunderland NHS Foundation Trust, Sunderland, SR4 7TP
13. Division of Immunology, Immunity to Infection and Respiratory Medicine, Faculty of Biology Medicine and Health, The University of Manchester, Manchester, M13 9PL
14. Critical Care Department, Northern Care Alliance NHS Foundation Trust, Salford Care Organisation, Greater Manchester, M6 8HD
15. Department of Respiratory Medicine, Royal Victoria Infirmary, Newcastle, NE1 4LP
16. Division of Anaesthesia, Department of Medicine, University of Cambridge, Cambridge, CB2 0QQ
17. Division of Immunology, Department of Pathology, University of Cambridge, Cambridge, CB2 1QP
18. JVF Intensive Care Unit, Addenbrooke's Hospital, Cambridge, CB2 0QQ
19. Department of Critical Care Medicine, Royal Victoria Infirmary, Newcastle, NE1 4LP

For peer review only

Abstract

Introduction

Sepsis is characterised by a dysregulated immune response to infection, with exaggerated pro-inflammatory and anti-inflammatory responses. A predominant immunosuppressive profile affecting both innate and adaptive immune responses is associated with increased hospital-acquired infection and reduced infection-free survival. While hospital-acquired infection leads to additional antibiotic use, the role of the immunosuppressive phenotype in guiding complex decisions, such as those affecting antibiotic stewardship, is uncertain. This study is a mechanistic sub-study embedded within a multi-centre clinical and cost-effectiveness trial of biomarker-guided antibiotic stewardship. This mechanistic study aims to determine the effect of sepsis-associated immunosuppression on the trial outcome measures.

Methods and analysis

RISC-sepsis is a prospective, multi-centre, exploratory, observational study embedded within the ADAPT-Sepsis trial. A sub-group of 180 participants with antibiotics commenced for suspected sepsis, enrolled in the ADAPT-sepsis trial, will be recruited. Blood samples will be collected on alternate days until day 7. At each time point, blood will be collected for flow cytometric analysis into cell preservation tubes. Immunophenotyping will be performed at a central testing hub by flow cytometry. The primary outcome measures are monocyte HLA-DR; neutrophil CD88; programmed cell death-1 (PD-1) on monocytes, neutrophils and T lymphocytes; and the percentage of regulatory T cells. Secondary outcome measures will link to trial outcomes from the ADAPT-Sepsis trial including antibiotic days; occurrence of hospital-acquired infection; and length of ICU- and hospital-stay.

Ethics and dissemination

Ethical approval has been granted (IRAS 209815) and the study registered with the ISRCTN (86837685). Study results will be disseminated by peer-reviewed publications, presentations at scientific meetings, and via patient and public participation groups and social media.

Strengths and weaknesses

- Multi-centre study recruiting a broad cohort of patients, representative of critically ill patients with sepsis in the UK.
- Centralised flow cytometry immunophenotyping using preservation methods allowing standardisation in the context of multi-centre recruitment.
- Embedded within a clinical trial, immunophenotypes will be linked to robust clinical outcomes.
- As an observational study RISC-sepsis will provide insights into the impact of sepsis-associated immunosuppression on a biomarker-guided antibiotic duration intervention but since it is not interventional, it will not offer a robust precision-medicine approach to antibiotic stewardship.

For peer review only

Introduction

Sepsis is a syndrome of life-threatening organ dysfunction secondary to a dysregulated immune response to an infection (1). Mortality from sepsis is high, with 31% of patients dying in hospital and a further 15% of survivors dying in the subsequent year (2,3). As a leading cause of death, there are an estimated 31.5 million cases of sepsis worldwide each year (4). Organ support in a critical care setting is often required and patients with sepsis account for a third of general adult critical care admissions in the UK (2).

The dysregulated immune response that characterises sepsis is a paradigm of both a pro-inflammatory and an anti-inflammatory response (5,6). While these immune states can occur simultaneously, a predominant immunosuppressive profile has been associated with hospital-acquired infections and mortality (7,8). While there is no single test for sepsis-associated immunosuppression, well-established leucocyte cell surface markers identify cellular dysfunction and are associated with adverse clinical outcomes. These dysfunctions are demonstrated across both the innate and adaptive immune responses with the most well-established markers being monocyte human leucocyte antigen-DR (mHLA-DR), neutrophil C5a receptor (CD88), percentage of regulator T cells and programmed cell death-1 (PD-1) (9–12). The persistence of these cellular abnormalities and the accumulation of dysfunctions increase the risk of adverse clinical outcomes (10,13–15).

While an immunosuppressive profile has been linked to poor clinical outcomes, the influence that it has in guiding complex clinical decision-making processes, such as those involved in antibiotic stewardship remains uncertain. Antibiotic treatment in sepsis may be life-saving but the overuse of antibiotics leads to the emergence of antimicrobial resistance (AMR) and adverse clinical outcomes (16–19). The optimal duration of antibiotic treatment in sepsis is unknown (20). Biomarker-guided antibiotic durations are not recommended for routine use in the UK (21) and the current evidence for effectiveness is regarded as low quality internationally (22). The ADAPT-Sepsis trial (ISRCTN47473244) is a UK National Institute for Health and Care Research (NIHR) Health Technology Assessment-commissioned clinical trial that will evaluate the clinical and cost effectiveness of procalcitonin (PCT) and C-reactive protein (CRP) protocols for biomarker-guided antibiotic duration decisions in a National Health Service (NHS) setting. This 3-arm trial of daily PCT monitoring, daily CRP monitoring or standard of care aims to determine whether the use of these biomarkers reduces total antibiotic use at 28-days (primary superiority outcome) while maintaining treatment safety of 28-day mortality (primary non-inferiority outcome). Investigations into the dynamics of CRP and PCT with mHLA-DR have shown that high levels of CRP or PCT are associated with low expression of mHLA-DR (23–25), suggesting CRP and PCT offer a window into the more complex dysregulation of the immune response.

This protocol outlines a mechanistic study embedded within the ADAPT-Sepsis trial. RISC-sepsis will immune phenotype patients using leukocyte surface biomarkers and dichotomise patients into two groups, 'sepsis-associated immunosuppression' and 'non-immunosuppressed' (definition below). We will then explore the effectiveness of the biomarker-guided antibiotic duration intervention and the impact on trial outcomes for patients with sepsis-associated immunosuppression.

Rationale

The ADAPT-Sepsis trial will include a heterogeneous population of adult patients with sepsis, including those with sepsis-induced immunosuppression. As a phenotype associated with hospital-

For peer review only

acquired infections and reduced infection-free survival, there may be heterogeneity in the effect of the biomarker-guided antibiotic duration intervention for patients with this phenotype. If biomarker-guided antibiotics are ineffective in patients with sepsis-associated immunosuppressed, then a greater degree of patient stratification may be required to ensure optimal antibiotic duration. Furthermore, if CRP and PCT levels fall over the same period that cellular defects resolve, this may give clinicians greater confidence in following biomarker-guided antibiotic durations.

Research question

Does sepsis-associated immunosuppression render a PCT/CRP biomarker-guided antibiotic duration intervention ineffective?

Hypothesis

Sepsis-associated immunosuppression will be associated with: longer duration of antibiotics, due to persistently raised PCT and CRP; more hospital-acquired infection; and more antibiotic treatment days.

Primary objective

To determine differences in ADAPT-sepsis trial outcomes between patients defined as 'sepsis-associated immunosuppressed' versus 'non-immunosuppressed'.

Secondary objective

To monitor changes in expression of leukocyte surface markers (primary outcome measures) over the time course of the ADAPT-Sepsis biomarker-guided antibiotic duration intervention period.

Methods and analysis

RISC-sepsis is a prospective, multi-centre, exploratory, observational study embedded within the ADAPT-Sepsis trial.

Population

We will include sequential patients who are enrolled into the ADAPT-sepsis trial within sites in the RISC-sepsis sub-study. Patients are included in the ADAPT-sepsis trial based on the following inclusion criteria:

1. Hospitalised adult patients at least 18 years of age
2. Up to 24 hours since initiation of antibiotics for suspected sepsis
3. Likely to remain hospitalised and requiring intravenous antibiotics for the next 72 hours
4. Requirement for critical care

Patients are excluded based on the following criteria:

1. More than 24 hours since receiving first empiric intravenous antibiotic treatments for suspicion of sepsis

For peer review only

2. Prolonged (greater than 21 days) antimicrobial therapy mandated (e.g. for endocarditis, cerebral/hepatic abscess, tuberculosis, osteomyelitis)
3. Severely immunocompromised (e.g. blood neutrophil count less than 0.5 x 10^9/L) not caused by sepsis
4. Any patient given, or anticipated to receive an IL-6 receptor inhibitor drug (e.g. tocilizumab or sarilumab) during their acute hospital admission
5. All treatment for suspected sepsis likely to be stopped within 24 hours of its initiation because of futility
6. Patient participation declined
7. Previously enrolled in ADAPT-Sepsis

Outcomes

The primary outcomes are markers of blood leukocyte function measured by flow cytometry. These primary markers are:

- Monocyte HLA-DR
- Neutrophil CD88
- Programmed cell death-1 on T lymphocytes (CD4-positive and CD8-positive), monocytes and neutrophils
- Percentage of regulatory T cells

Secondary outcome measures include outcome measures derived from the ADAPT-Sepsis trial including duration of antibiotics during the ADAPT-Sepsis intervention phase; total antibiotic days measured at 28 days; occurrence of hospital-acquired infection; and length of ICU- and hospital-stay. Secondary measures will also include PCT and CRP concentrations in serum.

Sampling and data collection

As a sub-study within the ADAPT-Sepsis trial, all RISC-sepsis procedures and sampling will occur within the trial. Sampling will occur during the ADAPT-Sepsis intervention period when blood is sampled daily. While additional blood samples are taken from patients for RISC-sepsis, there is no increase in number of sampling episodes. Patients will have blood sampled on alternate days up to 4 sampling points (days 1, 3, 5 and 7). Blood for flow cytometric analysis will be collected in Cyto-Chex (Streck, La Vista, USA) blood preservation tubes before shipping to a central testing hub (Newcastle University). Serum will be collected, frozen and stored at site until the end of the recruitment period.

Baseline clinical data and outcome data will be obtained from the ADAPT-sepsis data collection. Baseline data will include admission diagnosis, site of infection, acute physiology and chronic health evaluation (APACHE) II and sequential organ failure assessment (SOFA) scores, and sepsis bundle care elements in the first 24 hours. In addition, at each RISC-sepsis sampling point, the circulating white blood cell count and differential count will be recorded.

Blood sample analysis

Flow cytometric analysis will be performed on a single centralised (Newcastle University) BD FACSymphony A5 flow cytometer (Becton Dickinson Biosciences, San Jose, California, USA, from

For peer review only

here BDB). Daily internal quality control will be performed using Cytometry Setup and Tracking beads (BDB).

Leukocyte staining will be performed using antibody:fluorophore conjugates supplied by BDB. Samples will be processed according to a standard operating procedure.

At each time point, serum will be collected for measurement of PCT and CRP. These will be batched and processed by enzyme-linked immunosorbent assay (ELISA) at the end of the recruitment period.

Immune phenotyping

Cells will be identified based on light scatter properties and cellular markers: CD15++ neutrophils; CD14+ monocytes; CD3+ CD4+ T cells; CD3+ CD8+ T cells; and CD3+ CD4+ CD25+ CD127lo regulatory T cells. Expression of monocyte HLA-DR, neutrophil CD88 and PD-1 (CD279) on monocytes, neutrophils and T lymphocytes will be measured.

In addition, exploratory analyses will include: CD14+ CD16- classical monocytes; CD14+ CD16+ intermediate monocytes; CD14- CD16+ non-classical monocytes; CD3- CD19+ B cells; CD3- CD56+ NK cells; CD3- CD19- CD14- CD16- CD56- HLA-DR+ dendritic cells. In addition to expression of HLA-DR and CD279 in cell populations, CD274 (PD-L1) will also be measured.

Data will be assessed for evidence of batch effects and normalisation approaches will be used. Expert manual gating will be performed using a pre-defined gating standard operating procedure. We will report number and percentage of cell populations in blood and median fluorescent intensity of key markers.

Definition of sepsis-associated immunosuppression

We will define 'sepsis-associated immunosuppression' as two or more abnormalities (from low HLA-DR, low CD88, raised PD-1 or raised percentage regulatory T cells) that persist for 3 or more days (14,26). Previous reports have shown that the persistence of immune dysfunctions is associated with worse clinical outcome including increased hospital-acquired infections and death (10,13,15). The 'non-immunosuppressed' group will be patients that do not meet these criteria.

Sample size

The sample size is based on detection of a difference in antibiotic durations between the 'sepsis-associated immunosuppression' and 'non-immunosuppressed' groups. The 2016 Surviving Sepsis Campaign guidelines recommended 7-10 days of antibiotics for patients with sepsis (27). We have assumed that patients with sepsis-associated immunosuppression will have longer durations (10 days) and so with a standard deviation of 6 days (derived from the ADAPT-sepsis trial), then a sample of 180 patients is required to detect a difference of 3 days in antibiotic treatment between the 'sepsis-associated immunosuppressed' and 'non-immunosuppressed' patients with 90% power, a 5% alpha and a 5% withdrawal rate.

Data analysis plan

For peer review only

A formal analysis plan will be finalised and deposited on an open access platform before the database is locked.

For the primary objective, the time it takes for antibiotics to be stopped in patients during the ADAPT-sepsis biomarker-guided antibiotic phase will be summarised and the distribution of the outcome described for the 'sepsis-associated immunosuppression' and the 'non-immunosuppressed' groups. The mean number of antibiotic days will be compared between groups obtained from linear regression models and adjusted for confounding variables. In addition, each of the cellular markers will be assessed as a continuous outcome. The change in cellular markers between each time point will be correlated with the number of days on antibiotics using Spearman's correlation coefficient. One sample t-tests will be used to assess the changes between time points.

The trial outcomes of 28-day antibiotics, hospital-acquired infections and length of ICU- and hospital-stay will be summarised for the 'sepsis-associated immunosuppressed' and 'non-immunosuppressed' groups. Differences between groups will be assessed using linear regression for continuous variables and logistic regression for categorical variables. We will describe the difference between the groups using p-values, point estimates and 95% confidence intervals.

For the secondary objective, we will assess the difference in the PCT/CRP levels and cellular markers for patients who met the criteria for 'sepsis-associated immunosuppressed' and when immune profile recovered (i.e. became 'non-immunosuppressed'). Assessments will be captured over time, and we will compute a longitudinal analysis (taking time as a random effect) to assess the difference in the two groups.

Sub-group analyses:

Exploratory analysis will be carried out for the following sub-groups:

1. Patients receiving critical care for a medical *versus* a surgical cause of sepsis.
2. Patients receiving critical care because of trauma *versus* non-trauma.
3. Patients receiving critical care because of a community-acquired infection *versus* a hospital-acquired infection
4. Infection site: community-acquired pneumonia; hospital-acquired pneumonia; urinary tract infection; intra-abdominal infection; or positive blood culture.
5. Patients with septic shock, as defined by persistent hypotension requiring vasopressors to maintain a MAP $\geq$ 65 mmHg and a serum lactate of > 2 mmol/L (1).

Regression models will be used with 'sepsis-associated immunosuppressed' as an interaction term by sub-group for number of days on antibiotics.

Public and Patient Involvement

Members of the public and patients were involved through the Pathfinder group, a group of critical survivors and representatives in the North East of England. The Pathfinder group were involved in developing the research question and lay summary. Ongoing public and patient involvement is through the ADAPT-Sepsis Trial Management and Trial Steering Groups, which incorporate RISC-sepsis oversight, and have members of the Intensive Care Society's Patient and Relatives group.

For peer review only - http://bmjopen.bmj.com/site/about/guidelines.xhtml

Ethics and dissemination

As a sub-study, the protocol has been incorporated into the ADAPT-sepsis protocol and ethical approval has been granted by the South Central – Oxford C Research Ethics Committee (IRAS 209815). The study has been registered with the ISRCTN registry (86837685) and adopted by the UK National Institute for Health and Care Research (NIHR) Clinical Research Portfolio. The study is managed by the Warwick Clinical Trials Unit (WCTU) and Sponsored by The University of Manchester. Independent study oversight is provided by the ADAPT-Sepsis trial steering committee.

The study will be reported in line with the STROBE guidelines for observational studies (28). Findings will be disseminated by peer-reviewed publications and at national and international meetings. In addition, findings will be disseminated via public and patient groups and through social media.

Authors' contribution

The study was conceived by TPH, ACM, MSH, PD, TW, GB and AJS. TPH, ACM, MSH, PD, RMcM, DFMcA, TW, AJS, AR, GP and IMcC acquired research funding. Flow cytometry methods were developed by JS, LT, DFMcA, AF, AR, ACM, MSH, GB, VM and DMcD. The project is managed by HMcN, TE, PM and TPH. Statistical analysis will be performed by RL, KB and TPH. The draft manuscript was prepared by TPH, ACM, AJS and AF. All authors contributed to reviewing and editing the manuscript.

Funding statement

This study/project is funded by the NIHR Efficacy and Mechanism Evaluation (EME) (128374). The views expressed are those of the author(s) and not necessarily those of the NIHR or the Department of Health and Social Care. Regents for flow cytometric analysis are provided by Becton Dickinson Biosciences. The study is also supported by the MRC SHIELD consortium (MRNO2995X/1).

Competing interests statement

DFMcA is NIHR/MRC EME programme director and has previously sat on NIHR HTA funding committees.

All other authors report no competing interests in relation to this work.

References

1. Singer M, Deutschman CS, Seymour C, Shankar-Hari M, Annane D, Bauer M, et al. The third international consensus definitions for sepsis and septic shock (sepsis-3). JAMA. 2016;315(8):801–10.
2. Shankar-Hari M, Harrison DA, Rubenfeld GD, Rowan K. Epidemiology of sepsis and septic shock in critical care units: comparison between sepsis-2 and sepsis-3 populations using a national critical care database. Br J Anaesth. 2017;119(4):626–36.
3. Shankar-Hari M, Harrison DA, Ferrando-Vivas P, Rubenfeld GD, Rowan K. Risk Factors at

For peer review only

Index Hospitalization Associated With Longer-term Mortality in Adult Sepsis Survivors. JAMA Netw Open. 2019;2(5):e194900.

4. Fleischmann C, Scherag A, Adhikari NKJ, Hartog CS, Tsaganos T, Schlattmann P, et al. Assessment of Global Incidence and Mortality of Hospital-treated Sepsis. Current Estimates and Limitations. Am J Respir Crit Care Med. 2016;193(3):259–72.
5. van der Poll T, van de Veerdonk FL, Scicluna BP, Netea MG. The immunopathology of sepsis and potential therapeutic targets. Nat Rev Immunol. 2017;17(7):407–20.
6. Hotchkiss RS, Monneret G, Payen D. Immunosuppression in sepsis: a novel understanding of the disorder and a new therapeutic approach. Lancet Infect Dis. 2013;13(3):260–8.
7. Boomer JS, To K, Chang KC, Takasu O, Osborne DF, Walton AH, et al. Immunosuppression in patients who die of sepsis and multiple organ failure. JAMA. 2011;306(23):2594–605.
8. Pfortmueller CA, Meisel C, Fux M, Schefold JC. Assessment of immune organ dysfunction in critical illness: utility of innate immune response markers. Intensive Care Med Exp. 2017;5(1):49.
9. Guignant C, Lepape A, Huang X, Kherouf H, Denis L, Poitevin F, et al. Programmed death-1 levels correlate with increased mortality, nosocomial infection and immune dysfunctions in septic shock patients. Crit Care. 2011;15(2):R99.
10. Cheron A, Floccard B, Allaouchiche B, Guignant C, Poitevin F, Malcus C, et al. Lack of recovery in monocyte human leukocyte antigen-DR expression is independently associated with the development of sepsis after major trauma. Crit Care. 2010;14(6):R208.
11. Conway Morris A, Kefala K, Wilkinson TS, Dhaliwal K, Farrell L, Walsh T, et al. C5a mediates peripheral blood neutrophil dysfunction in critically ill patients. Am J Respir Crit Care Med. 2009;180(1):19–28.
12. Zhao Y, Jia Y, Li C, Fang Y, Shao R. The risk stratification and prognostic evaluation of soluble programmed death-1 on patients with sepsis in emergency department. Am J Emerg Med. 2018;36(1):43–8.
13. Bodinier M, Peronnet E, Brengel-Pesce K, Conti F, Rimmelé T, Textoris J, et al. Monocyte Trajectories Endotypes Are Associated With Worsening in Septic Patients. Front Immunol. 2021;12:5096.
14. Conway Morris A, Datta D, Shankar-Hari M, Stephen J, Weir CJ, Rennie J, et al. Cell-surface signatures of immune dysfunction risk-stratify critically ill patients: INFECT study. Intensive Care Med. 2018;44(5):627–35.
15. Drewry AM, Samra N, Skrupky LP, Fuller BM, Compton SM, Hotchkiss RS. Persistent lymphopenia after diagnosis of sepsis predicts mortality. Shock. 2014;42(5):383–91.
16. Chatterjee A, Modarai M, Naylor NR, Boyd SE, Atun R, Barlow J, et al. Quantifying drivers of antibiotic resistance in humans: a systematic review. Lancet Infect Dis. 2018;18(12):e368–78.
17. Bonnet V, Dupont H, Glorion S, Aupée M, Kipnis E, Gérard JL, et al. Influence of bacterial resistance on mortality in intensive care units: a registry study from 2000 to 2013 (IICU Study). J Hosp Infect. 2019;102(3):317–24.
18. Kett DH, Cano E, Quartin AA, Mangino JE, Zervos MJ, Peyrani P, et al. Implementation of guidelines for management of possible multidrug-resistant pneumonia in intensive care: an observational, multicentre cohort study. Lancet Infect Dis. 2011;11(3):181–9.
19. Tamma PD, Avdic E, Li DX, Dzintars K, Cosgrove SE. Association of Adverse Events With Antibiotic Use in Hospitalized Patients. JAMA Intern Med. 2017;177(9):1308.
20. Hellyer TP, Mantle T, McMullan R, Dark P. How to optimise duration of antibiotic treatment in patients with sepsis? BMJ. 2020;371:11–3.
21. NICE. Procalcitonin testing for diagnosing and monitoring sepsis (ADVIA Centaur BRAHMS

For peer review only

PCT assay, BRAHMS PCT Sensitive Kryptor assay, Elecsys BRAHMS PCT assay, LIAISON BRAHMS PCT assay and VIDAS BRAHMS PCT assay) DG18. 2015;

22. Evans L, Rhodes A, Alhazzani W, Antonelli M, Coopersmith CM, French C, et al. Surviving Sepsis Campaign: International Guidelines for Management of Sepsis and Septic Shock 2021. Crit Care Med. 2021;49(11):e1063–143.

23. Tschaikowsky K, Hedwig-Geissing M, Schiele A, Bremer F, Schywalsky M, Schüttler J. Coincidence of pro- and anti-inflammatory responses in the early phase of severe sepsis: Longitudinal study of mononuclear histocompatibility leukocyte antigen-DR expression, procalcitonin, C-reactive protein, and changes in T-cell subsets in septic and p. Crit Care Med. 2002;30(5):1015–23.

24. Cajander S, Rasmussen G, Tina E, Magnuson A, Söderquist B, Källman J, et al. Dynamics of monocytic HLA-DR expression differs between bacterial etiologies during the course of bloodstream infection. PLoS One. 2018;13(2):e0192883.

25. Almansa R, Martín S, Martin-Fernandez M, Heredia-Rodríguez M, Gómez-Sánchez E, Aragón M, et al. Combined quantification of procalcitonin and HLA-DR improves sepsis detection in surgical patients. Sci Rep. 2018;8(1):11999.

26. Shankar-Hari M, Datta D, Wilson J, Assi V, Stephen J, Weir CJ, et al. Early PREdiction of sepsis using leukocyte surface biomarkers: the ExPRES-sepsis cohort study. Intensive Care Med. 2018;44(11):1836–1848.

27. Rhodes A, Evans LE, Alhazzani W, Levy MM, Antonelli M, Ferrer R, et al. Surviving Sepsis Campaign: International Guidelines for Management of Sepsis and Septic Shock: 2016. Crit Care Med. 2017;45(3):486–552.

28. Vandenbroucke JP, Von Elm E, Altman DG, Gøtzsche PC, Mulrow CD, Pocock SJ, et al. Strengthening the Reporting of Observational Studies in Epidemiology (STROBE): Explanation and Elaboration. PLOS Med. 2007 Oct;4(10):e297.

For peer review only

BMJ Open

The Role of Immunosuppression in an antibiotic Stewardship intervention and its association with Clinical outcomes and antibiotic use: Protocol for an observational study (RISC-sepsis)

Journal:	*BMJ Open*
Manuscript ID	bmjopen-2022-068321.R2
Article Type:	Protocol
Date Submitted by the Author:	24-Nov-2022

Complete List of Authors:	Scott, Jonathan; Newcastle University, Translational and Clinical Research Institute<br>Trevi, Loredana; Newcastle University, Translational and Clinical Research Institute<br>McNeil, Hannah; University of Warwick, Clinical Trials Unit<br>Ewen, Tom; Newcastle University, Translational and Clinical Research Institute<br>Mawson, Phil; Newcastle University, Translational and Clinical Research Institute<br>McDonald, David; Newcastle University, Flow Cytometry Core Facility<br>Filby, Andrew; Newcastle University, Flow Cytometry Core Facility<br>Lall, Ranjit; University of Warwick, Warwick Clinical Trials Unit<br>Booth, Katie; University of Warwick, Warwick Clinical Trials Unit<br>Boschman, Gert; Becton Dickinson<br>Melkebeek, Vesna; Becton Dickinson<br>Perkins, Gavin; University of Warwick, Clinical Trials Unit<br>McMullan, Ronan; Royal Victoria Hospital, Department of Medical Microbiology; Queen's University Belfast, Wellcome Wolfson Institute for Experimental Medicine<br>McAuley, Daniel; Queen's University Belfast, Centre for Experimental Medicine; Belfast Health and Social Care Trust, Regional Intensive Care Unit<br>McCullagh, Iain J; Newcastle Upon Tyne Hospitals NHS Foundation Trust, Department of Perioperative medicine; Freeman Hospital, Department of Perioperative medicine<br>Walsh, Timothy; Edinburgh Royal Infirmary, Intensive Care Unit; The University of Edinburgh, The Queen's Medical Research Institute<br>Rostron, Anthony; South Tyneside and Sunderland NHS Foundation Trust, Integrated Critical Care Unit; Newcastle University, Translational and Clinical Research Institute<br>Shankar-Hari, Manu; University of Edinburgh Division of Clinical and Surgical Sciences, Centre for Inflammation Research; Edinburgh Royal Infirmary, Intensive Care Unit<br>Dark, Paul; University of Manchester, Division of Immunology; Salford Care Organisation, Critical Care Department<br>Simpson, A John; Newcastle University Institute of Cellular Medicine, Institute of Cellular Medicine, Medical School; Royal Victoria Infirmary, Department of Respiratory Medicine

	Conway-Morris, Andrew; University of Cambridge, Department of Medicine; Addenbrooke's Hospital, JVF Intensive Care Unit<br>Hellyer, Thomas P; Newcastle University, Translational and Clinical Research Institute; Royal Victoria Infirmary, Department of Critical Care Medicine
**Primary Subject Heading**:	Intensive care
Secondary Subject Heading:	Infectious diseases, Immunology (including allergy), Diagnostics
Keywords:	MICROBIOLOGY, INTENSIVE & CRITICAL CARE, INFECTIOUS DISEASES

SCHOLARONE™
Manuscripts

The Role of Immunosuppression in an antibiotic Stewardship intervention and its association with Clinical outcomes and antibiotic use: Protocol for an observational study (RISC-sepsis)

Jonathan Scott[1†], Loredana Trevi[1†], Hannah McNeil[2], Tom Ewen[1], Phil Mawson[1], David McDonald[3], Andrew Filby[3], Ranjit Lall[2], Katie Booth[2], Gert Boschman[4], Vesna Melkebeek[4], Gavin D Perkins[2,5], Ronan McMullan[6, 7], Danny McAuley[6, 8], Iain J McCullagh [1, 9], Timothy S Walsh[10, 11], Anthony Rostron[1, 12], Manu Shankar-Hari[10, 11], Paul Dark[13, 14], A John Simpson[1, 15], Andrew Conway Morris[16, 17,18], Thomas P Hellyer[1, 19*]

[†]These authors contributed equally

*Corresponding author, thomas.hellyer@newcastle.ac.uk

1. Translational and Clinical Research Institute, Faculty of Medical Sciences, Newcastle University, Newcastle-upon-Tyne, NE2 4HH
2. Warwick Clinical Trials Unit, Warwick Medical School, The University of Warwick, Coventry, CV4 7AL
3. Flow Cytometry Core Facility, Faculty of Medical Sciences, Newcastle University, Newcastle-upon-Tyne, NE2 4HH
4. Becton Dickinson, Erembodegem, Belgium
5. Critical Care Department, Birmingham Heartlands Hospital, Birmingham, B9 5SS
6. Wellcome Wolfson Institute for Experimental Medicine, Queens University Belfast, University Road, Belfast, BT7 1NN
7. Microbiology Department, Royal Victoria Hospital, Belfast, BT12 6BA
8. Regional Intensive Care Unit, Royal Victoria Hospital, Belfast Health and Social Care, Belfast, BT12 6BA
9. Department of Perioperative medicine, Critical care and Pain, Freeman Hospital, Newcastle-upon-Tyne, NE7 7DN
10. The Queen's Medical Research Institute, Edinburgh BioQuarter, Centre for Inflammation Research, Edinburgh, EH16 4TJ
11. Intensive Care Unit, Edinburgh Royal Infirmary, Edinburgh, EH16 4SA
12. Integrated Critical Care Unit, Sunderland Royal Hospital, South Tyneside and Sunderland NHS Foundation Trust, Sunderland, SR4 7TP
13. Division of Immunology, Immunity to Infection and Respiratory Medicine, Faculty of Biology Medicine and Health, The University of Manchester, Manchester, M13 9PL
14. Critical Care Department, Northern Care Alliance NHS Foundation Trust, Salford Care Organisation, Greater Manchester, M6 8HD
15. Department of Respiratory Medicine, Royal Victoria Infirmary, Newcastle, NE1 4LP
16. Division of Anaesthesia, Department of Medicine, University of Cambridge, Cambridge, CB2 0QQ
17. Division of Immunology, Department of Pathology, University of Cambridge, Cambridge, CB2 1QP
18. JVF Intensive Care Unit, Addenbrooke's Hospital, Cambridge, CB2 0QQ
19. Department of Critical Care Medicine, Royal Victoria Infirmary, Newcastle, NE1 4LP

For peer review only

Abstract

Introduction

Sepsis is characterised by a dysregulated immune response to infection, with exaggerated pro-inflammatory and anti-inflammatory responses. A predominant immunosuppressive profile affecting both innate and adaptive immune responses is associated with increased hospital-acquired infection and reduced infection-free survival. While hospital-acquired infection leads to additional antibiotic use, the role of the immunosuppressive phenotype in guiding complex decisions, such as those affecting antibiotic stewardship, is uncertain. This study is a mechanistic sub-study embedded within a multi-centre clinical and cost-effectiveness trial of biomarker-guided antibiotic stewardship. This mechanistic study aims to determine the effect of sepsis-associated immunosuppression on the trial outcome measures.

Methods and analysis

RISC-sepsis is a prospective, multi-centre, exploratory, observational study embedded within the ADAPT-Sepsis trial. A sub-group of 180 participants with antibiotics commenced for suspected sepsis, enrolled in the ADAPT-sepsis trial, will be recruited. Blood samples will be collected on alternate days until day 7. At each time point, blood will be collected for flow cytometric analysis into cell preservation tubes. Immunophenotyping will be performed at a central testing hub by flow cytometry. The primary outcome measures are monocyte HLA-DR; neutrophil CD88; programmed cell death-1 (PD-1) on monocytes, neutrophils and T lymphocytes; and the percentage of regulatory T cells. Secondary outcome measures will link to trial outcomes from the ADAPT-Sepsis trial including antibiotic days; occurrence of hospital-acquired infection; and length of ICU- and hospital-stay.

Ethics and dissemination

Ethical approval has been granted (IRAS 209815) and the study registered with the ISRCTN (86837685). Study results will be disseminated by peer-reviewed publications, presentations at scientific meetings, and via patient and public participation groups and social media.

Strengths and limitations of this study

- Multi-centre study recruiting a broad cohort of patients, representative of critically ill patients with sepsis in the UK.
- Centralised flow cytometry immunophenotyping using preservation methods allowing standardisation in the context of multi-centre recruitment.
- Embedded within a clinical trial, immunophenotypes will be linked to robust clinical outcomes.
- As an observational study RISC-sepsis will provide insights into the impact of sepsis-associated immunosuppression on a biomarker-guided antibiotic duration intervention but since it is not interventional, it will not offer a robust precision-medicine approach to antibiotic stewardship.

For peer review only

Introduction

Sepsis is a syndrome of life-threatening organ dysfunction secondary to a dysregulated immune response to an infection (1). Mortality from sepsis is high, with 31% of patients dying in hospital and a further 15% of survivors dying in the subsequent year (2,3). As a leading cause of death, there are an estimated 31.5 million cases of sepsis worldwide each year (4). Organ support in a critical care setting is often required and patients with sepsis account for a third of general adult critical care admissions in the UK (2).

The dysregulated immune response that characterises sepsis is a paradigm of both a pro-inflammatory and an anti-inflammatory response (5,6). While these immune states can occur simultaneously, a predominant immunosuppressive profile has been associated with hospital-acquired infections and mortality (7,8). While there is no single test for sepsis-associated immunosuppression, well-established leucocyte cell surface markers identify cellular dysfunction and are associated with adverse clinical outcomes. These dysfunctions are demonstrated across both the innate and adaptive immune responses with the most well-established markers being monocyte human leucocyte antigen-DR (mHLA-DR), neutrophil C5a receptor (CD88), percentage of regulator T cells and programmed cell death-1 (PD-1) (9–12). The persistence of these cellular abnormalities and the accumulation of dysfunctions increase the risk of adverse clinical outcomes (10,13–15).

While an immunosuppressive profile has been linked to poor clinical outcomes, the influence that it has in guiding complex clinical decision-making processes, such as those involved in antibiotic stewardship remains uncertain. Antibiotic treatment in sepsis may be life-saving but the overuse of antibiotics leads to the emergence of antimicrobial resistance (AMR) and adverse clinical outcomes (16–19). The optimal duration of antibiotic treatment in sepsis is unknown (20). Biomarker-guided antibiotic durations are not recommended for routine use in the UK (21) and the current evidence for effectiveness is regarded as low quality internationally (22). The ADAPT-Sepsis trial (ISRCTN47473244) is a UK National Institute for Health and Care Research (NIHR) Health Technology Assessment-commissioned clinical trial that will evaluate the clinical and cost effectiveness of procalcitonin (PCT) and C-reactive protein (CRP) protocols for biomarker-guided antibiotic duration decisions in a National Health Service (NHS) setting. This 3-arm trial of daily PCT monitoring, daily CRP monitoring or standard of care aims to determine whether the use of these biomarkers reduces total antibiotic use at 28-days (primary superiority outcome) while maintaining treatment safety of 28-day mortality (primary non-inferiority outcome). Investigations into the dynamics of CRP and PCT with mHLA-DR have shown that high levels of CRP or PCT are associated with low expression of mHLA-DR (23–25), suggesting CRP and PCT offer a window into the more complex dysregulation of the immune response.

This protocol outlines a mechanistic study embedded within the ADAPT-Sepsis trial. RISC-sepsis will immune phenotype patients using leukocyte surface biomarkers and dichotomise patients into two groups, ‘sepsis-associated immunosuppression’ and ‘non-immunosuppressed’ (definition below). We will then explore the effectiveness of the biomarker-guided antibiotic duration intervention and the impact on trial outcomes for patients with sepsis-associated immunosuppression.

Rationale

The ADAPT-Sepsis trial will include a heterogeneous population of adult patients with sepsis, including those with sepsis-induced immunosuppression. As a phenotype associated with hospital-

For peer review only

acquired infections and reduced infection-free survival, there may be heterogeneity in the effect of the biomarker-guided antibiotic duration intervention for patients with this phenotype. If biomarker-guided antibiotics are ineffective in patients with sepsis-associated immunosuppressed, then a greater degree of patient stratification may be required to ensure optimal antibiotic duration. Furthermore, if CRP and PCT levels fall over the same period that cellular defects resolve, this may give clinicians greater confidence in following biomarker-guided antibiotic durations.

Research question

Does sepsis-associated immunosuppression render a PCT/CRP biomarker-guided antibiotic duration intervention ineffective?

Hypothesis

Sepsis-associated immunosuppression will be associated with: longer duration of antibiotics, due to persistently raised PCT and CRP; more hospital-acquired infection; and more antibiotic treatment days.

Primary objective

To determine differences in ADAPT-sepsis trial outcomes between patients defined as 'sepsis-associated immunosuppressed' versus 'non-immunosuppressed'.

Secondary objective

To monitor changes in expression of leukocyte surface markers (primary outcome measures) over the time course of the ADAPT-Sepsis biomarker-guided antibiotic duration intervention period.

Methods and analysis

RISC-sepsis is a prospective, multi-centre, exploratory, observational study embedded within the ADAPT-Sepsis trial.

Population

We will include sequential patients who are enrolled into the ADAPT-sepsis trial within sites in the RISC-sepsis sub-study. Patients are included in the ADAPT-sepsis trial based on the following inclusion criteria:

1. Hospitalised adult patients at least 18 years of age
2. Up to 24 hours since initiation of antibiotics for suspected sepsis
3. Likely to remain hospitalised and requiring intravenous antibiotics for the next 72 hours
4. Requirement for critical care

Patients are excluded based on the following criteria:

1. More than 24 hours since receiving first empiric intravenous antibiotic treatments for suspicion of sepsis

For peer review only

2. Prolonged (greater than 21 days) antimicrobial therapy mandated (e.g. for endocarditis, cerebral/hepatic abscess, tuberculosis, osteomyelitis)
3. Severely immunocompromised (e.g. blood neutrophil count less than 0.5 x 10^9/L) not caused by sepsis
4. Any patient given, or anticipated to receive an IL-6 receptor inhibitor drug (e.g. tocilizumab or sarilumab) during their acute hospital admission
5. All treatment for suspected sepsis likely to be stopped within 24 hours of its initiation because of futility
6. Patient participation declined
7. Previously enrolled in ADAPT-Sepsis

Outcomes

The primary outcomes are markers of blood leukocyte function measured by flow cytometry. These primary markers are:

- Monocyte HLA-DR
- Neutrophil CD88
- Programmed cell death-1 on T lymphocytes (CD4-positive and CD8-positive), monocytes and neutrophils
- Percentage of regulatory T cells

Secondary outcome measures include outcome measures derived from the ADAPT-Sepsis trial including duration of antibiotics during the ADAPT-Sepsis intervention phase; total antibiotic days measured at 28 days; occurrence of hospital-acquired infection; and length of ICU- and hospital-stay. Secondary measures will also include PCT and CRP concentrations in serum.

Sampling and data collection

As a sub-study within the ADAPT-Sepsis trial, all RISC-sepsis procedures and sampling will occur within the trial. Sampling will occur during the ADAPT-Sepsis intervention period when blood is sampled daily. While additional blood samples are taken from patients for RISC-sepsis, there is no increase in number of sampling episodes. Patients will have blood sampled on alternate days up to 4 sampling points (days 1, 3, 5 and 7). Blood for flow cytometric analysis will be collected in Cyto-Chex (Streck, La Vista, USA) blood preservation tubes before shipping to a central testing hub (Newcastle University). Serum will be collected, frozen and stored at site until the end of the recruitment period.

Baseline clinical data and outcome data will be obtained from the ADAPT-sepsis data collection. Baseline data will include admission diagnosis, site of infection, acute physiology and chronic health evaluation (APACHE) II and sequential organ failure assessment (SOFA) scores, and sepsis bundle care elements in the first 24 hours. In addition, at each RISC-sepsis sampling point, the circulating white blood cell count and differential count will be recorded.

Blood sample analysis

Flow cytometric analysis will be performed on a single centralised (Newcastle University) BD FACSymphony A5 flow cytometer (Becton Dickinson Biosciences, San Jose, California, USA, from

For peer review only

here BDB). Daily internal quality control will be performed using Cytometry Setup and Tracking beads (BDB).

Leukocyte staining will be performed using antibody:fluorophore conjugates supplied by BDB. Samples will be processed according to a standard operating procedure.

At each time point, serum will be collected for measurement of PCT and CRP. These will be batched and processed by enzyme-linked immunosorbent assay (ELISA) at the end of the recruitment period.

Immune phenotyping

Cells will be identified based on light scatter properties and cellular markers: CD15++ neutrophils; CD14+ monocytes; CD3+ CD4+ T cells; CD3+ CD8+ T cells; and CD3+ CD4+ CD25+ CD127lo regulatory T cells. Expression of monocyte HLA-DR, neutrophil CD88 and PD-1 (CD279) on monocytes, neutrophils and T lymphocytes will be measured.

In addition, exploratory analyses will include: CD14+ CD16- classical monocytes; CD14+ CD16+ intermediate monocytes; CD14- CD16+ non-classical monocytes; CD3- CD19+ B cells; CD3- CD56+ NK cells; CD3- CD19- CD14- CD16- CD56- HLA-DR+ dendritic cells. In addition to expression of HLA-DR and CD279 in cell populations, CD274 (PD-L1) will also be measured.

Data will be assessed for evidence of batch effects and normalisation approaches will be used. Expert manual gating will be performed using a pre-defined gating standard operating procedure. We will report number and percentage of cell populations in blood and median fluorescent intensity of key markers.

Definition of sepsis-associated immunosuppression

We will define 'sepsis-associated immunosuppression' as two or more abnormalities (from low HLA-DR, low CD88, raised PD-1 or raised percentage regulatory T cells) that persist for 3 or more days (14, 26). Previous reports have shown that the persistence of immune dysfunctions is associated with worse clinical outcome including increased hospital-acquired infections and death (10,13,15). The 'non-immunosuppressed' group will be patients that do not meet these criteria.

Sample size

The sample size is based on detection of a difference in antibiotic durations between the 'sepsis-associated immunosuppression' and 'non-immunosuppressed' groups. The 2016 Surviving Sepsis Campaign guidelines recommended 7-10 days of antibiotics for patients with sepsis (27). We have assumed that patients with sepsis-associated immunosuppression will have longer durations (10 days) and so with a standard deviation of 6 days (derived from the ADAPT-sepsis trial), then a sample of 180 patients is required to detect a difference of 3 days in antibiotic treatment between the 'sepsis-associated immunosuppressed' and 'non-immunosuppressed' patients with 90% power, a 5% alpha and a 5% withdrawal rate.

Data analysis plan

For peer review only

A formal analysis plan will be finalised and deposited on an open access platform before the database is locked.

For the primary objective, the time it takes for antibiotics to be stopped in patients during the ADAPT-sepsis biomarker-guided antibiotic phase will be summarised and the distribution of the outcome described for the 'sepsis-associated immunosuppression' and the 'non-immunosuppressed' groups. The mean number of antibiotic days will be compared between groups obtained from linear regression models and adjusted for confounding variables. In addition, each of the cellular markers will be assessed as a continuous outcome. The change in cellular markers between each time point will be correlated with the number of days on antibiotics using Spearman's correlation coefficient. One sample t-tests will be used to assess the changes between time points.

The trial outcomes of 28-day antibiotics, hospital-acquired infections and length of ICU- and hospital-stay will be summarised for the 'sepsis-associated immunosuppressed' and 'non-immunosuppressed' groups. Differences between groups will be assessed using linear regression for continuous variables and logistic regression for categorical variables. We will describe the difference between the groups using p-values, point estimates and 95% confidence intervals.

For the secondary objective, we will assess the difference in the PCT/CRP levels and cellular markers for patients who met the criteria for 'sepsis-associated immunosuppressed' and when immune profile recovered (i.e. became 'non-immunosuppressed'). Assessments will be captured over time, and we will compute a longitudinal analysis (taking time as a random effect) to assess the difference in the two groups.

Sub-group analyses:

Exploratory analysis will be carried out for the following sub-groups:

1. Patients receiving critical care for a medical *versus* a surgical cause of sepsis.
2. Patients receiving critical care because of trauma *versus* non-trauma.
3. Patients receiving critical care because of a community-acquired infection *versus* a hospital-acquired infection
4. Infection site: community-acquired pneumonia; hospital-acquired pneumonia; urinary tract infection; intra-abdominal infection; or positive blood culture.
5. Patients with septic shock, as defined by persistent hypotension requiring vasopressors to maintain a MAP ≥ 65 mmHg and a serum lactate of > 2 mmol/L (1).

Regression models will be used with 'sepsis-associated immunosuppressed' as an interaction term by sub-group for number of days on antibiotics.

Study duration

The study was planned to commence in April 2020 but due to the COVID-19 pandemic, the start date was delayed until August 2020. Further delays were experienced during the optimisation of flow cytometry and site set up phases. Recruitment commenced in May 2022 and is ongoing. The study is planned to complete in April 2023.

Public and Patient Involvement

For peer review only

Members of the public and patients were involved through the Pathfinder group, a group of critical survivors and representatives in the North East of England. The Pathfinder group were involved in developing the research question and lay summary. Ongoing public and patient involvement is through the ADAPT-Sepsis Trial Management and Trial Steering Groups, which incorporate RISC-sepsis oversight, and have members of the Intensive Care Society's Patient and Relatives group.

Ethics and dissemination

As a sub-study, the protocol has been incorporated into the ADAPT-sepsis protocol and ethical approval has been granted by the South Central – Oxford C Research Ethics Committee (IRAS 209815). The study has been registered with the ISRCTN registry (86837685) and adopted by the UK National Institute for Health and Care Research (NIHR) Clinical Research Portfolio. The study is managed by the Warwick Clinical Trials Unit (WCTU) and Sponsored by The University of Manchester. Independent study oversight is provided by the ADAPT-Sepsis trial steering committee.

The study will be reported in line with the STROBE guidelines for observational studies (28). Findings will be disseminated by peer-reviewed publications and at national and international meetings. In addition, findings will be disseminated via public and patient groups and through social media.

Authors' contribution

The study was conceived by TPH, ACM, MSH, PD, TW, GB and AJS. TPH, ACM, MSH, PD, RMcM, DFMcA, TW, AJS, AR, GP and IMcC acquired research funding. Flow cytometry methods were developed by JS, LT, DFMcA, AF, AR, ACM, MSH, GB, VM and DMcD. The project is managed by HMcN, TE, PM and TPH. Statistical analysis will be performed by RL, KB and TPH. The draft manuscript was prepared by TPH, ACM, AJS and AF. All authors contributed to reviewing and editing the manuscript.

Funding statement

This study/project is funded by the NIHR Efficacy and Mechanism Evaluation (EME) (128374). The views expressed are those of the author(s) and not necessarily those of the NIHR or the Department of Health and Social Care. Regents for flow cytometric analysis are provided by Becton Dickinson Biosciences. The study is also supported by the MRC SHIELD consortium (MRNO2995X/1).

Competing interests statement

DFMcA is NIHR/MRC EME programme director and has previously sat on NIHR HTA funding committees.

All other authors report no competing interests in relation to this work.

References

1. Singer M, Deutschman CS, Seymour C, Shankar-Hari M, Annane D, Bauer M, et al. The third

For peer review only

international consensus definitions for sepsis and septic shock (sepsis-3). JAMA. 2016;315(8):801–10.

2. Shankar-Hari M, Harrison DA, Rubenfeld GD, Rowan K. Epidemiology of sepsis and septic shock in critical care units: comparison between sepsis-2 and sepsis-3 populations using a national critical care database. Br J Anaesth. 2017;119(4):626–36.
3. Shankar-Hari M, Harrison DA, Ferrando-Vivas P, Rubenfeld GD, Rowan K. Risk Factors at Index Hospitalization Associated With Longer-term Mortality in Adult Sepsis Survivors. JAMA Netw Open. 2019;2(5):e194900.
4. Fleischmann C, Scherag A, Adhikari NKJ, Hartog CS, Tsaganos T, Schlattmann P, et al. Assessment of Global Incidence and Mortality of Hospital-treated Sepsis. Current Estimates and Limitations. Am J Respir Crit Care Med. 2016;193(3):259–72.
5. van der Poll T, van de Veerdonk FL, Scicluna BP, Netea MG. The immunopathology of sepsis and potential therapeutic targets. Nat Rev Immunol. 2017;17(7):407–20.
6. Hotchkiss RS, Monneret G, Payen D. Immunosuppression in sepsis: a novel understanding of the disorder and a new therapeutic approach. Lancet Infect Dis. 2013;13(3):260–8.
7. Boomer JS, To K, Chang KC, Takasu O, Osborne DF, Walton AH, et al. Immunosuppression in patients who die of sepsis and multiple organ failure. JAMA. 2011;306(23):2594–605.
8. Pfortmueller CA, Meisel C, Fux M, Schefold JC. Assessment of immune organ dysfunction in critical illness: utility of innate immune response markers. Intensive Care Med Exp. 2017;5(1):49.
9. Guignant C, Lepape A, Huang X, Kherouf H, Denis L, Poitevin F, et al. Programmed death-1 levels correlate with increased mortality, nosocomial infection and immune dysfunctions in septic shock patients. Crit Care. 2011;15(2):R99.
10. Cheron A, Floccard B, Allaouchiche B, Guignant C, Poitevin F, Malcus C, et al. Lack of recovery in monocyte human leukocyte antigen-DR expression is independently associated with the development of sepsis after major trauma. Crit Care. 2010;14(6):R208.
11. Conway Morris A, Kefala K, Wilkinson TS, Dhaliwal K, Farrell L, Walsh T, et al. C5a mediates peripheral blood neutrophil dysfunction in critically ill patients. Am J Respir Crit Care Med. 2009;180(1):19–28.
12. Zhao Y, Jia Y, Li C, Fang Y, Shao R. The risk stratification and prognostic evaluation of soluble programmed death-1 on patients with sepsis in emergency department. Am J Emerg Med. 2018;36(1):43–8.
13. Bodinier M, Peronnet E, Brengel-Pesce K, Conti F, Rimmelé T, Textoris J, et al. Monocyte Trajectories Endotypes Are Associated With Worsening in Septic Patients. Front Immunol. 2021;12:5096.
14. Conway Morris A, Datta D, Shankar-Hari M, Stephen J, Weir CJ, Rennie J, et al. Cell-surface signatures of immune dysfunction risk-stratify critically ill patients: INFECT study. Intensive Care Med. 2018;44(5):627–35.
15. Drewry AM, Samra N, Skrupky LP, Fuller BM, Compton SM, Hotchkiss RS. Persistent lymphopenia after diagnosis of sepsis predicts mortality. Shock. 2014;42(5):383–91.
16. Chatterjee A, Modarai M, Naylor NR, Boyd SE, Atun R, Barlow J, et al. Quantifying drivers of antibiotic resistance in humans: a systematic review. Lancet Infect Dis. 2018;18(12):e368–78.
17. Bonnet V, Dupont H, Glorion S, Aupée M, Kipnis E, Gérard JL, et al. Influence of bacterial resistance on mortality in intensive care units: a registry study from 2000 to 2013 (IICU Study). J Hosp Infect. 2019;102(3):317–24.
18. Kett DH, Cano E, Quartin AA, Mangino JE, Zervos MJ, Peyrani P, et al. Implementation of guidelines for management of possible multidrug-resistant pneumonia in intensive care: an

For peer review only

observational, multicentre cohort study. Lancet Infect Dis. 2011;11(3):181–9.

19. Tamma PD, Avdic E, Li DX, Dzintars K, Cosgrove SE. Association of Adverse Events With Antibiotic Use in Hospitalized Patients. JAMA Intern Med. 2017;177(9):1308.

20. Hellyer TP, Mantle T, McMullan R, Dark P. How to optimise duration of antibiotic treatment in patients with sepsis? BMJ. 2020;371:11–3.

21. NICE. Procalcitonin testing for diagnosing and monitoring sepsis (ADVIA Centaur BRAHMS PCT assay, BRAHMS PCT Sensitive Kryptor assay, Elecsys BRAHMS PCT assay, LIAISON BRAHMS PCT assay and VIDAS BRAHMS PCT assay) DG18. 2015;

22. Evans L, Rhodes A, Alhazzani W, Antonelli M, Coopersmith CM, French C, et al. Surviving Sepsis Campaign: International Guidelines for Management of Sepsis and Septic Shock 2021. Crit Care Med. 2021;49(11):e1063–143.

23. Tschaikowsky K, Hedwig-Geissing M, Schiele A, Bremer F, Schywalsky M, Schüttler J. Coincidence of pro- and anti-inflammatory responses in the early phase of severe sepsis: Longitudinal study of mononuclear histocompatibility leukocyte antigen-DR expression, procalcitonin, C-reactive protein, and changes in T-cell subsets in septic and p. Crit Care Med. 2002;30(5):1015–23.

24. Cajander S, Rasmussen G, Tina E, Magnuson A, Söderquist B, Källman J, et al. Dynamics of monocytic HLA-DR expression differs between bacterial etiologies during the course of bloodstream infection. PLoS One. 2018;13(2):e0192883.

25. Almansa R, Martín S, Martin-Fernandez M, Heredia-Rodríguez M, Gómez-Sánchez E, Aragón M, et al. Combined quantification of procalcitonin and HLA-DR improves sepsis detection in surgical patients. Sci Rep. 2018;8(1):11999.

26. Shankar-Hari M, Datta D, Wilson J, Assi V, Stephen J, Weir CJ, et al. Early PREdiction of sepsis using leukocyte surface biomarkers: the ExPRES-sepsis cohort study. Intensive Care Med. 2018;44(11):1836–1848.

27. Rhodes A, Evans LE, Alhazzani W, Levy MM, Antonelli M, Ferrer R, et al. Surviving Sepsis Campaign: International Guidelines for Management of Sepsis and Septic Shock: 2016. Crit Care Med. 2017;45(3):486–552.

28. Vandenbroucke JP, Von Elm E, Altman DG, Gøtzsche PC, Mulrow CD, Pocock SJ, et al. Strengthening the Reporting of Observational Studies in Epidemiology (STROBE): Explanation and Elaboration. PLOS Med. 2007 Oct;4(10):e297.

For peer review only
